# Annotations

**Published:** 1887-08-06

**Authors:** 


### Annotations.

Medical Men and Infectious Diseases.

Alderman Belsey and the Mayor of Rochester are angry with the medical men of that town, because the authorities have been at the expense of providing a hospital for infectious diseases, which hospital, on account of the action of the medical men, might as well be shut up. The Mayor and the Alderman believe that the local doctors, for purposes of their own, have among themselves a more or less recognised agreement to "boycott" the infectious hospital. The conductors of the *Chatham Observer*, with laudable energy, have considered it their duty to inquire into the matter, with the object of giving the public the advantage of the doctors' view of the question. The doctors admit that the hospital has been very little used, and that probably the fact is due to their not having reported cases of fever as they have occurred. They deny, however, that there has been any agreement or "boycotting" in the matter. Each medical man has apparently taken his own way, and it has happened that all the ways have tended in the same direction. They say, in effect, that it is no business of theirs to act as officials of the city council. They further aver that in few cases would the friends of patients be willing to have it known that particular diseases existed in their houses. They evidently hold the opinion that if a general system of compulsory reporting prevailed, people would defer calling in medical men at the early stage of a complaint, and thus much valuable time would be lost, the chances of recovery diminished, and the possibility of the diseases spreading intensified. Moreover, they considered that in many houses isolation can be rendered as effective as in the hospital. The Mayor very foolishly hinted that the city council would compel the faculty to report cases occurring in the city. At present the city council have no more power to compel doctors to do any such thing than they have to compel the rooks to cease from cawing, or the donkeys of the parish from braying. No doubt the compulsory registration of infectious diseases would be of advantage to the public, but in the meantime there is no statutory provision for registration, and until such provision is made, the doctors are entirely within their right, and are well advised to act each according to his own view of what is best in particular cases.

Infectious Hospitals.

Some of the people of Salford are up in arms against a proposal to build an Infectious Diseases Hospital in their locality. Popular opinion being what it is on the subject of those diseases, we cannot wonder at their action. At the same time they are fighting against shadows. A collection of people suffering from all kinds of dangerous fevers a hundred years ago was one thing: such a collection of persons to-day is quite another thing. Then, the merest elements of sanitary hospital science were unknown or despised. Now, the utmost details and even refinements of that science are carried out with the most rigid exactness and completeness. A hospital for infectious diseases is a ten times safer place to live in than an ill-drained, ill-cleansed, and badly aerated street or house. The opponents of infectious hospitals should ask themselves how often, or rather how seldom, the resident doctors and nurses catch infectious diseases at those institutions. It is safe to say that if those officials be carefully selected, worked moderately, and have their general health well maintained by suitable diet, sleep, and other conditions of bodily vigour, such a thing as catching a fever in the hospital is hardly ever known. It is practically certain that a well-managed fever hospital emits no effluvia of any kind into the surrounding neighbourhood which can be the least injurious to man, woman, or child. A little knowledge in this instance is a very troublesome thing.

To avoid Infection.

Our great-grandfathers used to live in terror of evil spirits which destroyed them with pestilence, sent a murrain on their flocks and herds, got into the churns when the butter was in process, and played a thousand other dangerous or merely ridiculous freaks. What the unnamed spirits and demons were to them, contagion, infection, germs, bacilli, bacteria, micrococci, and innumerable other dreadful names and creatures are to us. Many of us live in mortal terror of we know not what. Air is doubtful, milk is dangerous, water is fatal. We sniff and deodorise, disinfect and purify, filter and boil, from morning till night. On the whole we do right. An enemy misunderstood is always dreaded, and it cannot be said that the public generally have an intelligent understanding of the various causes of what are called zymotic diseases. Most people know, however, that air and water are the two principal media of infection. Pure air and pure water mean comparative safety. Air is by no means so dangerous as water. Very much less harm is done by foul smells than is generally thought. If the water be good in quality there is little fear of catching or spreading infectious diseases. In summer especially the two watchwards in every house should be "Filter and boil." If people would but remember that open doors and windows, good drains, disinfectants, filtration and boiling, comprise a good part of the ten commandments of hygiene, and if they would do their best to pay obedience to this simple sanitary decalogue, they might go to sleep with a fair degree of safety, and with minds free from hygienic cares. Common-sense is the best guide when infection has to be avoided, and if all reasonable precautions have been taken, a wise and good man puts his trust in God and fears not. Fear is the most fruitful source of infection, as epidemics have frequently demonstrated all the world over.

